# One-pot preparation of substituted pyrroles from α-diazocarbonyl compounds

**DOI:** 10.3762/bjoc.4.45

**Published:** 2008-11-28

**Authors:** Fernando de C da Silva, Mauricio G Fonseca, Renata de S Rianelli, Anna C Cunha, Maria C B V de Souza, Vitor F Ferreira

**Affiliations:** 1 Universidade Federal Fluminense, Departamento de Química Orgânica, Instituto de Química, Outeiro de São João Baptista, CEP 24020-141, Niterói, RJ, Brazil.

**Keywords:** diazocarbonyl, dihydrofuran, one-pot synthesis, pyrrole

## Abstract

In this work an efficient one-pot synthesis of substituted pyrroles **7a**–**n** is described, which involves the in situ formation of dihydrofurans ethyl 5-butoxy-2-methyl-4,5-dihydrofuran-3-carboxylate (**4**), 1-(5-butoxy-2-methyl-4,5-dihydrofuran-3-yl)ethanone (**5**) and 5-butoxy-4,5-dihydrofuran-3-carbaldehyde (**6**) followed by reaction with primary amines.

## Introduction

The pyrrole unit [1] occurs in many interesting classes of compounds such as pharmaceutical agents [2–5], conducting polymers [6–7], molecular optics [8–11], electronics [12], gas sensors for organic compounds [13], and as building blocks in many physiologically interesting natural products, such as alkaloids [14]. The classical methods of constructing pyrrole ring system include mainly Knorr or Paal–Knorr syntheses, which have been summarized in a wide number of review articles [15–19]. Due to these multiple uses and varieties of biological activities, the synthesis of this ring system has been subject of intense investigation. Danks [20] developed a high yield Paal–Knorr method of synthesis of pyrroles by the reaction between hexane-2,5-dione and primary amines under microwave irradiation. Other processes including several clay-mediated synthetic variations of these classical methods also have been reported for preparing pyrroles in equal or better yields [21]. In 2001 [22] and 2004 [23], Banik and co-workers, by using the same protocol developed by Danks, reported the synthesis of pyrroles using montmorillonite KSF in a solvent-free process accelerated by microwave irradiation. More recently, Yadav and co-workers [24] described an efficient protocol for the synthesis of sugar derived optically active di-pyrrolyl and bis-indolyl alkanols that are important for the synthesis of porphyrins, using montmorillonite KSF as catalyst. The search for short procedures for the synthesis of highly functionalized pyrrole derivatives is still desirable [25–27].

In the next years the organic synthetic chemists will have more demanding tasks, which include the search of products that can be manufactured in environmentally acceptable ways with minimum consumption of energy and abundant raw materials (e.g. biomass). The new reactions must maintain a favorable ecological balance to be acceptable by society. In many books, the definition of the ideal synthesis agrees with several demanding tasks mentioned before in such way that the target molecules should be made from readily available starting materials in one simple, safe, environmentally acceptable operation. Additionally, they also should proceed quickly, in quantitative yield, with high atom economy, and be more convergent than one or two-component reactions.

α-Diazocarbonyl compounds have a long history of useful applications in organic chemistry. They are easily prepared from readily accessible precursors and can be used in a wide variety of chemical transformations [28]. Indeed, we prepared several pyrroles from diazocarbonyl compounds in two steps [29] employing dihydrofurans. However, these latter compounds were difficult to prepare, isolate and transform into the pyrroles [30]. In this manuscript, we report the preparation of pyrroles from diazodicarbonyl compounds in a one-pot reaction.

## Results and Discussion

**Scheme 1:**
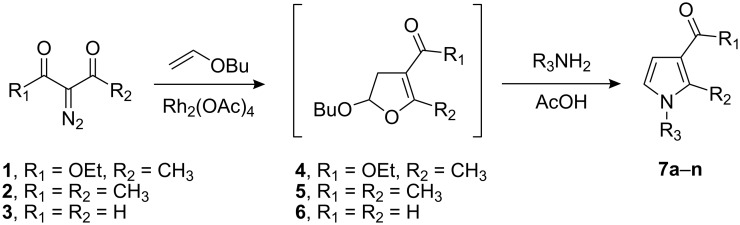
One-pot synthesis of pyrrole derivatives.

The substituted pyrroles **7a**–**n** were prepared according to the synthetic pathways described in Scheme 1. α-Diazocarbonyl compounds ethyl 2-diazoacetoacetate (**1**), 3-diazopentane-2,4-dione (**2**) and diazomalonaldehyde (**3**) were treated with a catalytic quantity of rhodium(II) acetate in the presence of butyl vinyl ether to produce the corresponding 3-carbonyl-dihydrofurans **4**–**6**. The rhodium catalyzed reaction of the α-diazocarbonyl compounds **1**–**3** was monitored by TLC chromatography. Evaporation of the solvents at the end of these reactions followed by purification of crude residues by column chromatography led to the pyrroles **7a**–**n** in moderate to good yields. Their structures were confirmed mainly based on their ^13^C and ^1^H NMR spectral data which are depicted in the experimental section.

Since the reactivities of the diazo compounds are different there were variations in the reaction times (see Table 1). The reaction of the dihydrofuran intermediates **4**, **5** and **6** with excess of primary amines in the presence of glacial acetic acid afforded the corresponding substituted pyrroles **7a**–**n** in moderate to good yields (Table 1). Slightly different reactivity between **4**, **5** and **6** was observed since the former reaction involves a nucleophilic attack to vinylogous carbonyls of 3-carbonyl-dihydrofurans.

**Table 1:** Substituted pyrroles (**7a**–**n**) prepared by this methodology.

Entry	7a–n	R_1_	R_2_	R_3_	t (h)	Yield (%)

1	**7a**	OEt	Me	Benzyl [29]	52	72
2	**7b**	OEt	Me	Decyl [29]	60	79
3	**7c**	OEt	Me	Cyclohexyl [29]	72	74
4	**7d**	OEt	Me	Isopropyl [31]	76	21
5	**7e**	OEt	Me	Butyl	48	69
6	**7f**	Me	Me	Decyl [29]	44	66
7	**7g**	Me	Me	Benzyl [29]	61	73
8	**7h**	Me	Me	Cyclohexyl [29]	56	64
9	**7i**	Me	Me	Isopropyl	54	23
10	**7j**	Me	Me	Butyl [29]	51	62
11	**7k**	H	H	Benzyl [30]	48	86
12	**7l**	H	H	Butyl [32]	48	83
13	**7m**	H	H	Propyl [33]	48	85
14	**7n**	H	H	H [34]	24	65

In general, the yields were dependent basically on the reactivity of the diazocompounds. In fact, diazocompound **3** led to higher yields and lower reaction times than **1** or **2** (entries 11–14). Most of the amines had little influence on the reaction yields, with the exception of isopropylamine that led to the pyrroles in lower yields (entries 4 and 9), probably due to steric hindrance of the methyl groups. In some experiments the products were obtained in a high degree of purity (entries 11 and 14). Additionally, pyrrole **7k**, in our previous work [30], was obtained in 16% yield and by using this methodology was obtained in higher yield (86%, entry 11).

## Conclusion

In summary, this one-pot methodology for the synthesis of substituted pyrroles from α-diazocarbonyl compounds is a very straightforward route to construct variously substituted compounds of this class starting from readily available precursors.

## Experimental

Analytical grade solvents were used. Butyl vinyl ether was freshly distilled before being used. Column chromatography was performed on silica gel 60 (Merck 70–230 mesh). Infrared spectra were recorded on a Perkin-Elmer 1420 spectrophotometer. NMR spectra were recorded with a Varian Unity Plus 300 spectrometer, operating at 300 MHz (^1^H) and 75 MHz (^13^C), with tetramethylsilane as the internal standard. Ethyl 2-diazoacetoacetate (**1**) [35], 3-diazopentane-2,4-dione (**2**) [36], and diazomalonaldehyde (**3**) [37] were prepared following the procedures described in the literature. Purified samples were used for measuring physical constants and spectral data. High-resolution mode TOF-ESIMS mass spectra were obtained with Hewlett Packard 5985 instrument.

### General procedure

A solution of the diazodicarbonyl compound (**1**–**3**, 2 mmol) in 5 mL of freshly distilled butyl vinyl ether was slowly added by a syringe pump, at a rate of 1.0 mL/h, to a stirred suspension of dirhodium tetraacetate (0.2 mmol) in 5.0 mL of the same butyl vinyl ether as solvent, under a nitrogen atmosphere. The stirring was continued until the disappearance of the diazodicarbonyl compound followed by the addition of the appropriate amine (4 mmol) and of 0.2 mL of glacial acetic acid. The mixture was stirred for the total time described in the Table 1. The solvent was removed under reduced pressure leading to a residue, which was purified by chromatography column on silica gel, using a gradient mixture of hexane/chloroform or chloroform/acetone as the eluent. For the compound **7n** (entry 14), the butyl vinyl ether solution was saturated with ammonia and stirred for 24 h.

**1-Butyl-2-methyl-1H-pyrrole-3-carboxylic acid ethyl ester (7e)**. It was obtained as a yellow oil. IR ν_max_/cm^−1^: 2595, 2873, 1701, 1464; ^1^H NMR (300 MHz, CDCl_3_) δ 0.93 (3H, t, J = 7.2 Hz), 1.35 (3H, t, J = 7.2 Hz), 1.45 (2H, sept, J = 7.2 Hz), 1.68 (2H, quint, J = 7.5 Hz), 2.47 (3H, s), 3.84 (2H, t, J = 7.5 Hz), 4.22 (2H, q, J = 7.2 Hz), 6.53 (1H, d, J = 3.0 Hz), 6.55 (1H, d, J = 3.0 Hz); ^13^C NMR (75 MHz, CDCl_3_) δ 10.5 (CH_3_), 13.7 (C-4′), 14.1 (OCH_2_CH_3_), 19.3 (C-3′), 32.8 (C-2′), 45.8 (C-1′), 58.9 (OCH_2_CH_3_), 109.5 (C-4), 112.0 (C-3), 119.1 (C-5), 135.3 (C-2), 165.0 (C=O). HRMS calcd for C_12_H_20_NO_2_ [M+H]^+^: 210.1494, found 210.2959.

**1-(1-Isopropyl-2-methyl-1H-pyrrol-3-yl)-ethanone (7i)**. It was obtained as a brown oil. IR ν_max_/cm^−1^: 2925, 2871, 1738; ^1^H NMR (300 MHz, CDCl_3_) δ 1.25 (3H, t, J = 7.0 Hz), 1.27 (3H, t, J = 7.0 Hz), 2.40 (3H, s), 2.53 (3H, s), 3.67–3.80 (1H, m), 6.48 (1H, d, J = 3.0 Hz), 6.53 (1H, d, J = 3.0 Hz); ^13^C NMR (75.0 MHz, CDCl_3_) δ 11.0 (CH_3_C_2_), 28.7 (CH_3_C=O), 32.4 and 32.7 (C-2′), 46.1 (C-1′), 109.3 (C-4), 119.1 (C-5), 120.5 (C-3), 134.1 (C-2), 195.4 (C=O). HRMS calcd for C_10_H_16_NO [M+H]^+^: 166.1232, found 166.2433.
